# UniProt and Mass Spectrometry-Based Proteomics—A 2-Way Working Relationship

**DOI:** 10.1016/j.mcpro.2023.100591

**Published:** 2023-06-08

**Authors:** E.H. Bowler-Barnett, J. Fan, J. Luo, M. Magrane, M.J. Martin, S. Orchard, The UniProt Consortium

**Affiliations:** 1 European Molecular Biology Laboratory, European Bioinformatics Institute (EMBL-EBI), Wellcome Genome Campus, Hinxton, United Kingdom; 2 Protein Information Resource, Georgetown University Medical Center, Washington, District of Columbia, USA; 3 Protein Information Resource, University of Delaware, Ammon-Pinizzotto Biopharmaceutical Innovation Building, Newark, Delaware, USA; 4 SIB Swiss Institute of Bioinformatics, Centre Medical Universitaire, Geneva, Switzerland

**Keywords:** UniProt, protein sequence database, protein function, complete proteomes, sequence identifiers

## Abstract

The human proteome comprises of all of the proteins produced by the sequences translated from the human genome with additional modifications in both sequence and function caused by nonsynonymous variants and posttranslational modifications including cleavage of the initial transcript into smaller peptides and polypeptides. The UniProtKB database (www.uniprot.org) is the world’s leading high-quality, comprehensive and freely accessible resource of protein sequence and functional information and presents a summary of experimentally verified, or computationally predicted, functional information added by our expert biocuration team for each protein in the proteome. Researchers in the field of mass spectrometry–based proteomics both consume and add to the body of data available in UniProtKB, and this review highlights the information we provide to this community and the knowledge we in turn obtain from groups *via* deposition of large-scale datasets in public domain databases.

## Highlights

• High-quality, comprehensive, free resource of protein sequence and function.
• Reference sets of whole-proteome sequence, including human, made available.
• Includes isoforms, postprocessed chains, PTMs, variants, and functional domains.
• Imports and displays peptide and PTM identifications from public domain repositories.
• Access via website, API or ftp, download data in multiple community data formats.

## Graphical Abstract

**Figure:**
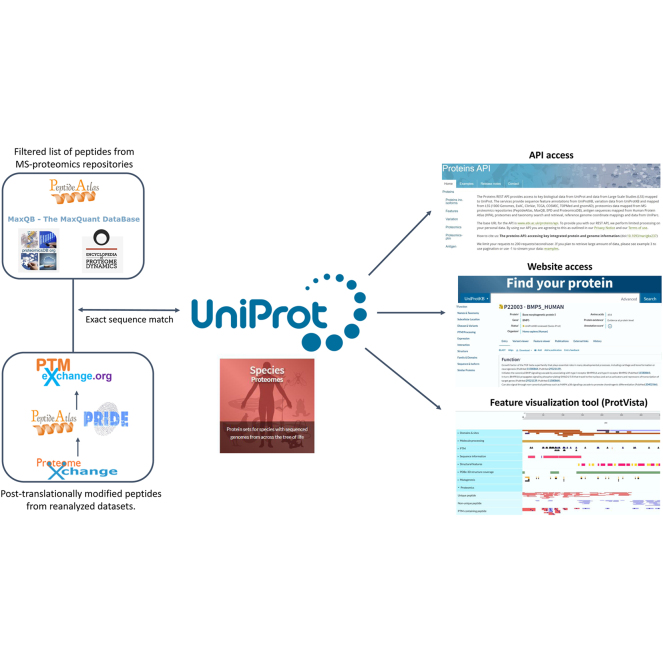

## In Brief

UniProt, the world’s leading high-quality, comprehensive, and freely accessible resource of protein sequence and functional information, is used by the global mass spectrometry–based proteomics community to map peptide identifications to proteins. The database supplies details of protein sequence features which may affect their function, such as posttranslation modifications. UniProt in turn takes data which researchers publish or deposit in public domain databases and uses this to increase the information content of relevant entries.

Mass spectrometry (MS)-based proteomics researchers aim to isolate, identify, and functionally characterize the protein profile of a cell, tissue, or organism of interest. Scientists may be interested in understanding how this profile changes as cells develop, age, respond to changes in external environmental conditions, or how the application of a xenobiotic, such as a drug, changes cellular behavior at the molecular level. In order for researchers to be able to determine the protein complement of a biological sample, they need to be able to map peptide or protein sequences to a reference resource, which not only enables the required identifications but also links these to the relevant organism and provides details of protein nomenclature, function, and additional sequence features such as posttranslational modifications (PTMs) and functional domains. The UniProt data resource (1) provides a complete compendium of all known protein sequence data linked to a summary of the experimentally verified, or computationally predicted, functional information about that protein and is the most widely used protein sequence database employed by the proteomics community. This review is aimed at enabling proteomics scientists to not only download the optimal set of sequences for use in their search engine(s) but also to subsequently access as much information on their identified proteins as possible. It also describes how MS data are used to inform and improve UniProt records and how we plan to extend that relationship moving forwards.

## What does UniProt Provide for the MS Community?

Sequence database search engines identify peptides from tandem mass spectra *via* comparison to a reference protein sequence database (2). The selection of which reference sequence database to use is therefore critical to the eventual output of the search engine algorithm and the results of the study. Database sequence coverage is of paramount importance, in that a search engine cannot identify peptides from proteins that are not present in the selected reference dataset. However, the protein sequence database should also enable the user to manage their search space, *i.e*., restrict the number of possible peptides against which a spectrum is searched, as too large a search space affects search engine speed, specificity, and sensitivity. Two of the most commonly used techniques to enable this are restricting the sequence selection to only one per gene and/or ignoring potential peptide modification by PTMs; however, both of these decisions require the sacrifice of information. The UniProt Knowledgebase (UniProtKB) (1) is increasingly the database of choice for the MS community, and here we explain the reasons behind this, how UniProtKB has been structured to serve proteomics use cases, and how users can access additional data to enable a more complete analysis of their biological and biomedical datasets.

## Sequence Annotation and Coverage

UniProtKB consists of reviewed UniProtKB/Swiss-Prot entries to which data have been added by our expert biocuration team and unreviewed UniProtKB/TrEMBL entries that are annotated by automated systems. While, for a limited number of species including Human, *Saccharomyces cerevisiae,* and *Escherichia coli*, there is a single reviewed entry for every known protein coding gene, for most species complete proteome coverage can only be achieved through a combination of reviewed and unreviewed entries. Reviewed entries contain the sequences of all the protein products encoded by one gene, each with a unique identifier. This includes protein isoforms expressed by that gene along with any shorter protein chain/peptide generated from the parent transcript. Isoforms are defined as sequences that can be generated from the same gene by a single or combination of biological events (alternative promoter usage, alternative splicing, alternative initiation, and ribosomal frameshifting). Isoforms yet to be manually integrated are maintained in unreviewed UniProtKB/TrEMBL entries but can be viewed in the corresponding reviewed entry on the website as a result of automated gene-centric mappings. To create a UniProtKB/Swiss-Prot entry, UniProt curators firstly examine the protein sequence and correct, where necessary, for errors caused by sequence annotation issues such as erroneously selected initiation sites or frameshifts. They identify peptide chains generated by posttranslational cleavage of the parent transcript. These chains may be associated with protein location and activation and are generated by the removal of a signal or transit peptide or be bioactive peptides with defined biological activities. Positional features described within the UniProt entry, such as binding sites, PTMs, and amino acid variants, are mapped to a selected representative (canonical) sequence which is the entry displayed by default on the website and in most download formats. All sequences (canonical and isoforms) are distributed in FASTA format as described in the ‘Accessing data’ section.

The main source of protein sequences in UniProt is the translation of nucleic acid sequence submissions to the International Nucleotide Sequence Database Collaboration (INSDC) source databases (3). Increasingly, these consist of the protein content of completely sequenced genomes, *i.e.*, a proteome and are supplemented by proteomes sequenced and/or annotated by groups such as Ensembl (4), the National Center for Biotechnology Information RefSeq (5), VectorBase (6) and WormBase ParaSite (7). Viral proteomes are manually checked and verified and periodically added to the database. UniProt also contains partial proteomes of many thousands of additional organisms. To enable researchers to evaluate proteome completeness and expected gene content, we provide the BUSCO V4 (Benchmarking Universal Single-Copy Orthologs) score (8) for vertebrate, arthropod, fungal, and prokaryotic proteomes. This score identifies complete, duplicated, fragmented, and potentially missing genes by comparison to a defined set of near-universal single copy orthologs (9). We also provide the results of the ‘Complete Proteome Detector’, an in-house algorithm, which statistically evaluates the completeness and quality of each proteome by directly comparing it to those of a group of at least three closely taxonomically related species. The Complete Proteome Detector classifies each proteome as either ‘standard’, ‘close to standard’, or an ‘outlier’, according to protein count *versus* the standard distribution of protein count expected for completeness in comparison to a cluster of closely related organisms (https://www.uniprot.org/help/assessing_proteomes). We additionally supply the assessment of the genome assembly status imported from the source of the genome assembly and annotation (*e.g*., Ensembl or RefSeq).

A subset of these proteomes are then selected as reference proteomes, by either community choice or computational methods based on sequence clustering by similarity, to represent a spectrum of well-studied model organisms and other organisms of interest for biomedical research and phylogeny. Proteomes can be searched and downloaded from a specific section of the website (https://www.uniprot.org/proteomes/). The most commonly accessed proteome is that of human, which is described in more detail below.

### The Human Proteome in UniProt

A first draft of the human proteome was completed in UniProtKB in 2008 and announced at the eighth Siena ‘From genome to proteome’ meeting (10). Since then, the content of this key proteome has been continually refreshed and updated with new sequences, additional sequence features and a wealth of functional data. UniProt has been collating human sequences since the first release of the original Swiss-Prot database in 1987, well in advance of the publication of the human genome, therefore many proteins have had to be retro-fitted to the current genome build (GRCh38 from the Genome Reference Consortium). The choice of canonical sequence representing the protein product of each human gene has been selected in collaboration with the CCDS (11) and MANE (12) projects, and since release 2022_01, UniProtKB contains cross-references to both projects. UniProtKB contains additional proteins, for which a corresponding gene does not exist in the current reference genome. so is a larger dataset that might be anticipated. In addition to proteins encoded by a gene simply not represented in the genome(s) sequenced as part of the original human genome project but directly submitted to the sequences databases *via* INSDC, these include some microproteins/peptides, retrotransposable elements, and sequences which may eventually be proven to be nontranslated pseudogenes. Work is currently ongoing in collaboration with the Ensembl, RefSeq, Human Pangenome, and HGNC teams to enable as near concurrence between genome, transcriptome, and proteome as possible.

**Fig. 1:**
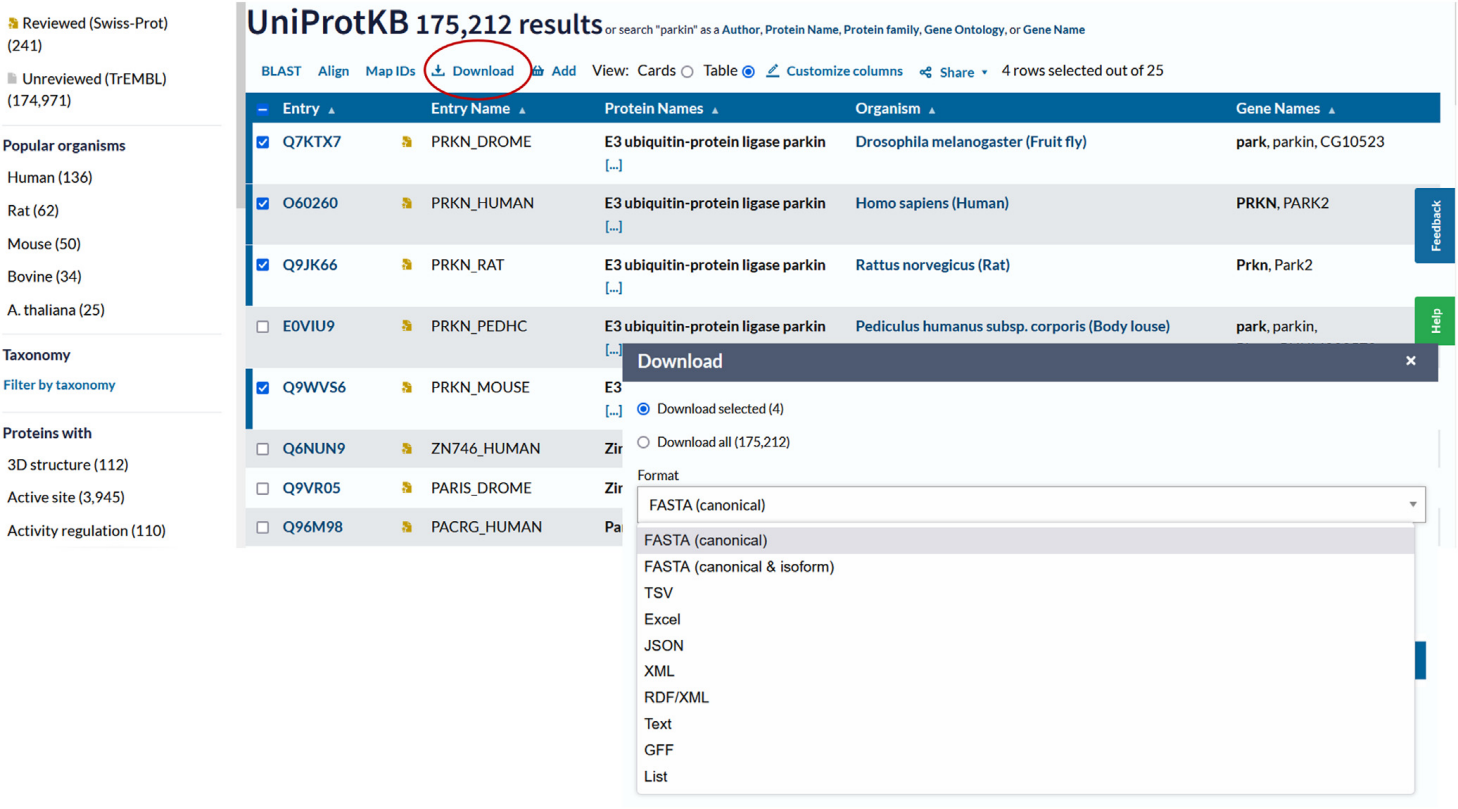
**Search results for ‘Parkin’ on****UniProt.org****.** Using the download option (*red circle*) allows users to download selected entries in a range of formats (see insert).

Different use cases require different assemblies of all the sequences available in the UniProtKB human proteome (Table 1). As previously stated, the UniProtKB/Swiss-Prot human proteome contains one representative (canonical) sequence for each currently known human protein-coding gene. More than 50% of these 20,000 entries contain manually reviewed alternative isoforms described within the entry, including sequences derived from alternative promoter usage, alternative splicing, alternative initiation, and ribosomal frameshifting. These reviewed isoforms sequences are made available together with the canonical sequences as a FASTA download file accessible from the website. In order to identify probable additional isoforms, a pipeline has also been established to import predicted human isoform sequences into UniProtKB/TrEMBL from the Ensembl database. Isoforms which were already existing in UniProtKB have mapped to the equivalent sequence in Ensembl (requiring 100% identity over 100% of the length of the two sequences) and are included as part of the proteome (*via* a link to the *Homo sapiens* proteome ID:UP000005640). Ensembl sequences that are absent from UniProtKB are imported into UniProtKB/TrEMBL. These entries are flagged as part of the proteome and have an Ensembl cross-reference. On the website they can be viewed as part of the UniProt reviewed entry for that gene and are described as “Computationally mapped potential isoform sequences”. Both canonical and all isoform sequences are available for download (Fig. 1). Ensembl-derived isoform sequences will only be added to the manually reviewed set when there is sufficient experimental evidence of their existence and potentially also functional data. Additional sequences imported into UniProt from the INSDC are available in UniProtKB/TrEMBL—these are not tagged as part of the reference proteome and may be redundant to sequences which are. However, valuable additional information may be available in these entries, such as relevant publications or novel protein coding genes. For example, recent publications have identified a number of small open reading frames originating from known ncRNAs (13, 14, 15), and these have been added to the human proteome set by manual curation into UniProtKB/Swiss-Prot.

**Table 1:** The human proteome in UniProtKB

Sequence set	No of sequences (2023_02)	Website query	Download
Canonical sequences	20,407	proteome:up000005640 AND reviewed:true	Website - FASTA (Canonical), Tab-separated, XML. RDF/XML,Text, GFF, JSON
aEntries containing additional reviewed isoforms	10,647	proteome:up000005640 AND (cc_ap:∗) (identifies entries containing reviewed isoforms)	Website - FASTA (Canonical + isoforms)
Number of reviewed isoforms	22,095		www.uniprot.org/help/downloads - FASTA
Additional unreviewed isoforms	62,085	proteome:up000005640 AND reviewed:false	Website - FASTA Tab-separated, XML. RDF/XML,Text, GFF
Complete proteome (Reviewed entries + unreviewed isoforms)	82,492	proteome:up000005640	Website - FASTA Tab-separated, XML. RDF/XML,Text, GFF
Total proteome (Canonical sequences + reviewed isoforms + unreviewed isoforms)	104,587	proteome:up000005640	Website - FASTA (download FASTA (canonical + isoform))
Additional UniProtKB/TrEMBL entries	125,361	(taxonomy_id:9606) AND (reviewed:false) NOT (proteome:up000005640)	Website - FASTA Tab-separated, XML. RDF/XML,Text, GFF

a Query gives entries containing reviewed isoforms, not individual entries for each isoform.

## Sequence Identifiers

Proteomic scientists require sequence identifiers to be stable and to conform to the FAIR principles (16, 17) of being Findable, Accessible, Interoperable, and Reusable. Stable identifiers mean that datasets can be analyzed over a period of time without the generated protein list apparently altering significantly, and spectra can be reanalyzed to find genuinely new proteins, not a known protein with a new identifier. In UniProtKB, stable, alphanumeric identifiers are issued at the sequence level and remain associated with the protein sequence if merged from an unreviewed into a reviewed entry or if a reviewed entry is demerged into two or more separate entries. An accession number is only deleted when the entry to which it was assigned has been removed from UniProtKB, for example, if a gene model prediction is deleted, in which case both accession number and sequence are retained in the UniProt Archive (UniParc) where they can be searched and accessed. Each isoform is characterized by a unique identifier, which is composed of the primary accession number of the corresponding canonical entry, followed by a dash and a number. Every posttranslationally cleaved protein chain/peptide is identified by a 3-letter prefix, PRO, separated by an underscore from a 6 to 10-digit number.

**Fig. 2:**
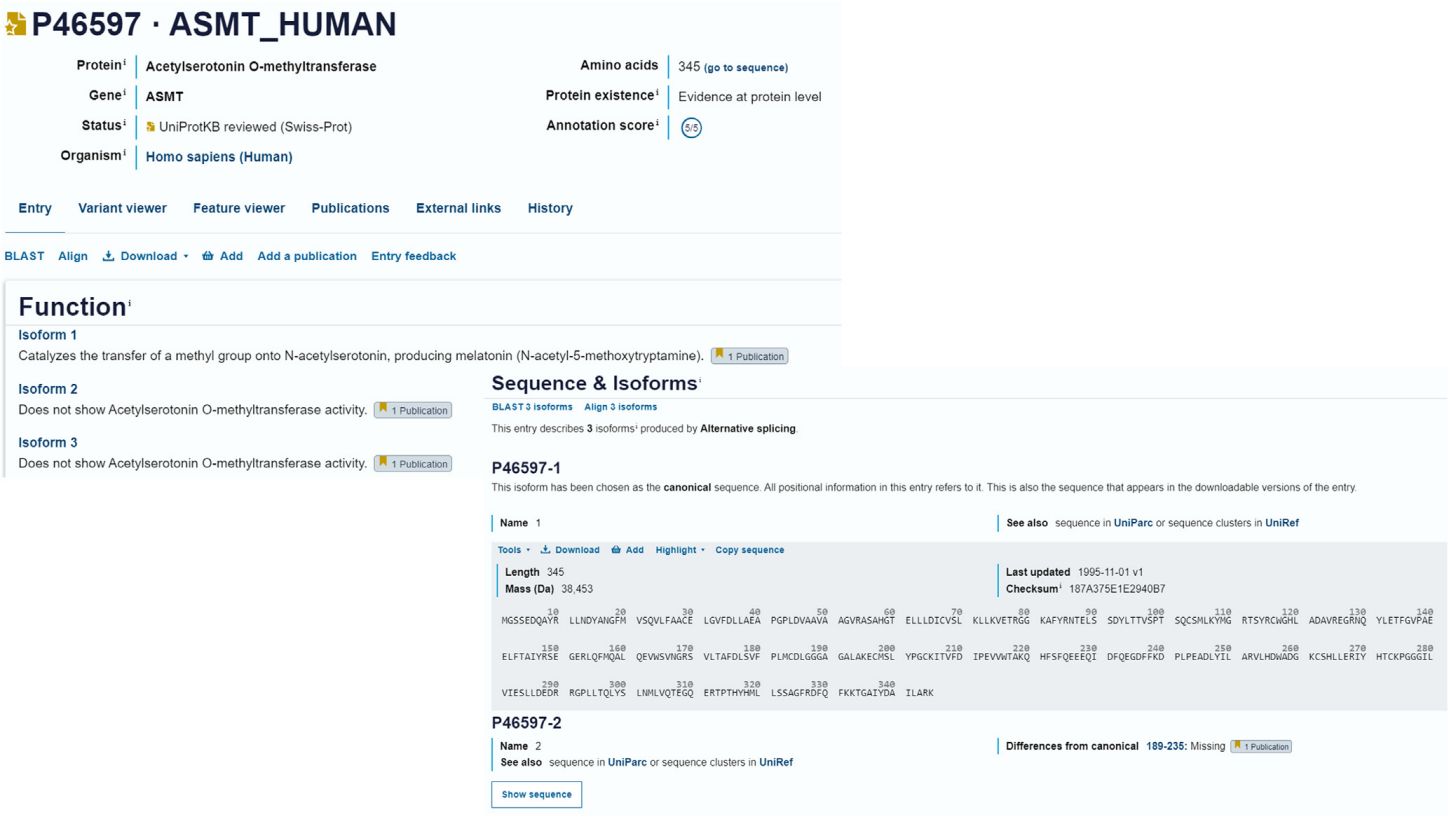
**In the sequence and isoforms subsection of the entry page, isoform amino acid sequences can be downloaded in a range of formats, and sequences can be aligned against one another for users to analyze their differences**. Where there is evidence of isoform-specific functions, and these are described separately in the Function comment of the protein entry (https://www.uniprot.org/uniprotkb/P46597). In this view, users may also access the ‘History’ of the changes made to each entry.

It should be noted that changes may be made to an underlying sequence, for example a change in the selected N-terminal methionine start point of a protein sequence or the switching of a single amino acid shown as the variant, usually as part on the ongoing efforts to reconcile the sequences displayed in the different sequence databases. Changes in the canonical sequence are indicated by a change in the version number, and this can be seen on the website in the ‘Sequence & isoforms’ section and is displayed in the FASTA download. If a different isoform is selected as the canonical sequence, its unique isoform identifier will remain associated with it. Proteomics researchers re-analyzing data after a period of time may, as a result, see a change in the peptides identified for a protein, but earlier versions of the entries, with the original entry canonical sequence displayed, may be accessed from the ‘History’ of each entry, *e.g.*, https://www.uniprot.org/uniprotkb/P04637/history (see Fig. 2 for the direct link from the website for each protein entry) if they need to understand this in more detail.

Users may have used a sequence database other than UniProtKB to make their initial peptide to protein identifications and mappings and may subsequently wish to map to UniProtKB to access the richness of data within this resource. UniProt provides an accession number mapping service (https://www.uniprot.org/id-mapping/) which enables users to convert between identifiers from an increasing number of resources to download the generated identifier list or simply to access a batch of UniProt entries to download or work with on the website.

## Sequence Features

Sequence features describe regions or sites of interest in the protein sequence, such as PTMs, binding sites, enzyme active sites, or local secondary structures. These features may be manually added by expert biocurators as a result of interpreting experimental evidences from peer-reviewed publications, may be the result of large-scale experiments such as MS-based proteomics, or may be computational predictions from resources such as the InterPro member databases. Each sequence annotation consists of a "feature key", the amino acid position(s) and a short, largely free-text description. Moving forward, the free-text descriptions will increasingly be mapped to ontology/controlled vocabulary terms to enhance the machine-readability of these data. As a first step toward this goal, the UniProt biocurators have replaced free-text descriptions of biologically relevant ligands which are crucial to protein function, including activators, inhibitors, cofactors, and substrates, with stable unique identifiers from the ChEBI (Chemical Entities of Biological Interest) ontology (18) making knowledge of ligands and their binding sites in UniProtKB easier to search and access (19).

Historically, large-scale datasets and most computational predictions have been mapped to the UniProt reviewed entry, rather than integrated into the downloadable version of the entry, as it was not trivial for the user to differentiate between manually reviewed and automatically generated data. However, the adoption by UniProtKB of evidence tags sourced from the Evidence and Conclusion Ontology (20) now makes it possible to discriminate between data sources, so these data may, in the future, be merged into UniProt records when it is of satisfactory quality.

### Posttranslational Modifications

**Fig. 3:**
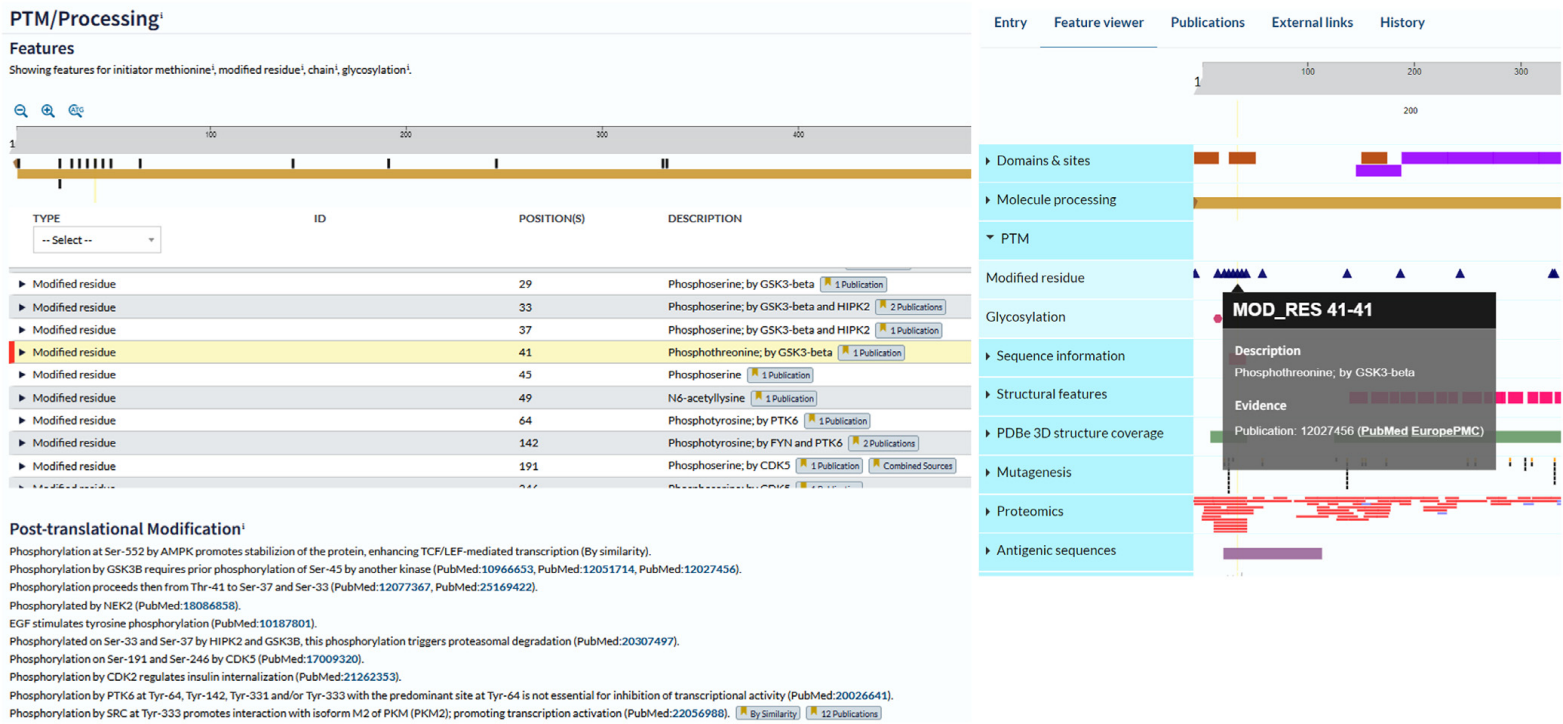
**Protein entry view, PTM/Processing subsection showing PTM residues mapped to the amino acid sequence of human CTNNB1 (****https://www.uniprot.org/uniprotkb/P35222****), with further detail available in the information table.** Additional PTM-related information is shown in free text format. The Feature Viewer displays the PTM track with expanded modified residue information for residue 41. PTM, posttranslational modification.

The presence of chemical modifications, added enzymatically to the protein backbone at various points during the life-cycle of a protein, can alter the function of a molecule, change its conformation and/or its subcellular location, and affect its stability and longevity. PTMs can provide a rapid mechanism for changing function, such as switching an enzyme activity "on" and "off" or causing a protein complex to disassemble. UniProt expert curators have mapped literature-described PTMs to protein sequences for many years, not only capturing details of the amino acid position and the chemical change (Fig. 3) but also, when known, the effect of the PTM on protein function and, when appropriate, also the identity of enzyme which makes the modification (Fig. 3). Literature evidence for the existence of PTMs may include antibody or point mutation studies where the PTM has effectively been prevented from forming (*e.g*., a Tyr to Phe mutation) or mimicked (*e.g*., a Ser to Glu mutation to mimic phosphorylation of the serine residue), in addition to studies by MS. Less common modification, where such tools are not available, may therefore currently be underrepresented in the database. These manually annotated PTMs have been used as a gold-standard set by MS proteomics researchers to assess the efficacy of their own pipelines to identify PTMs on a large scale. Users may access additional information on specific PTMs by using the cross references to specialist resources supplied in many entries.

### Domains, Sites, and Regions

**Fig. 4:**
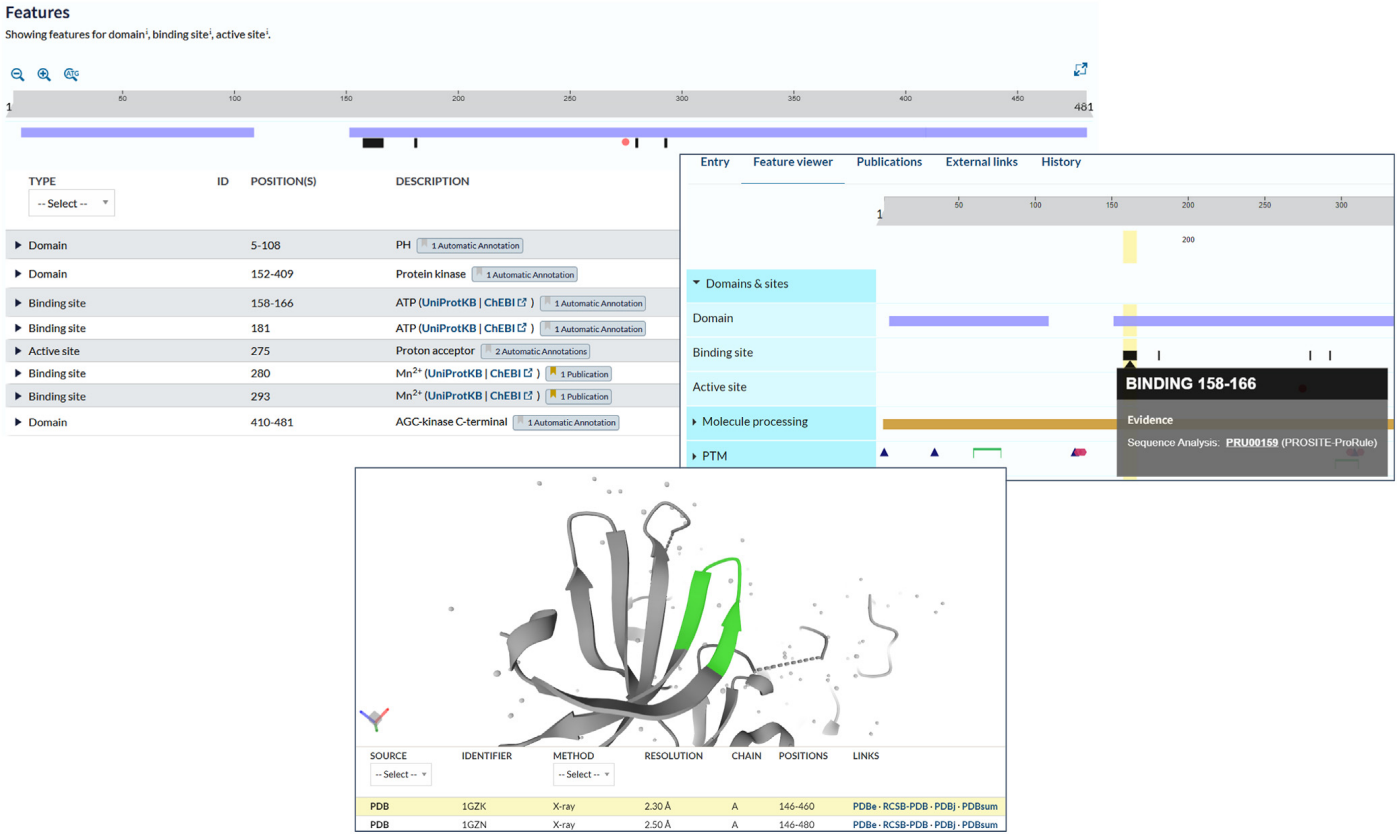
**Protein entry view, function subsection showing domains, sites, and regions mapped to the amino acid sequence of human AKT2 (****https://www.uniprot.org/uniprotkb/P31751****) with further details available in the information table**. *Insert right* shows the Feature Viewer with Domains & sites track with expanded information shown for a binding region. *Insert bottom* shows the 3D structure prediction for this protein with binding region highlighted.

The presence of conserved sets of amino acids in related protein sequences can give information about protein function, its location in the cell, for example membrane topology, and additional molecules to which they may bind, such as metals or cofactors (Fig. 4). In UniProtKB entries, these regions are usually initially predicted by tools such as InterProScan (21), with additional confirmation by an expert curator matching the region to functional data, conservation across species, and 3D structural information in reviewed UniProtKB entries.

### Variants and Mutations

**Fig. 5:**
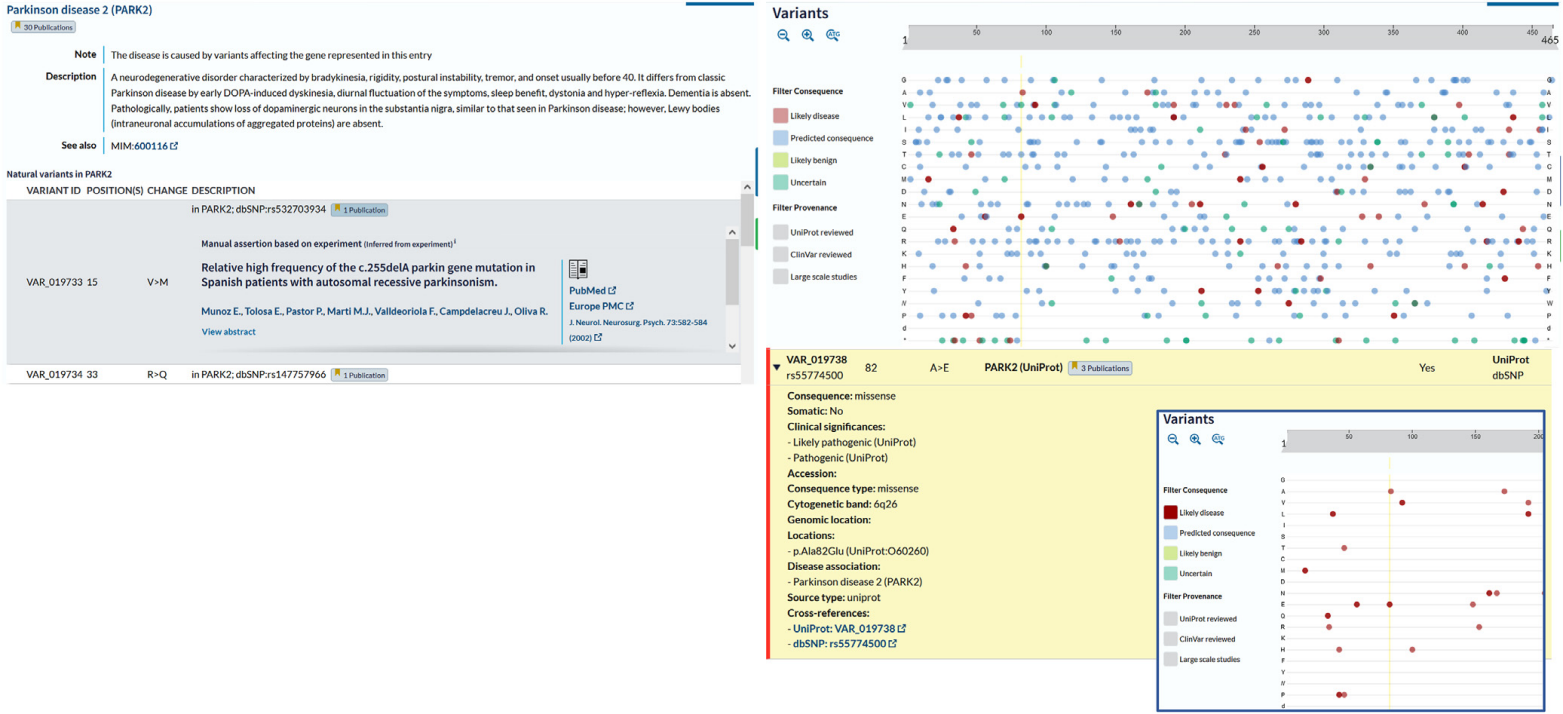
**Protein entry view, disease and variants subsection showing OMIM-derived disease description and associated variants**. Graphical plot of variants, plotted according to amino acid change consequence and position on the protein chain. Selection of a particular variant highlights detailed information in the table which is expandable for further information. Insert shows extract of variants filtered by consequence ‘Likely disease’.

UniProt curators capture detail on individual genetic variants, including RNA editing events, capturing the amino acid change, the name of the variant (or allele), when available, and the effect(s) of the variation on the protein, cell, or complete organism. Variant interpretation and classification are in line with the American College of Medical Genetics guidelines and additional recommendations by the ClinGen sequence variant interpretation workgroup (22). Similarly, the effects of individual, or sets of, experimentally induced mutations in the protein sequence are described. These mutated sites often provide valuable insights into understanding the mechanisms of protein function or may validate other data such as providing support for potential PTM sites. Human disease-linked variants are generally germline changes involved in Mendelian disease and linked to phenotypes described in the OMIM database (Fig. 5).

## Protein Nomenclature

While lists of accession numbers may be an efficient way of storing the results of a proteomics experiment, they tell the researcher nothing about the function of a protein and do not enable the grouping of members of the same protein family. UniProt curators are responsible for ensuring that proteins are consistently named, thus enabling literature searching and entry retrieval. A good protein name is unique, unambiguous, can be attributed to orthologs from other species, and follows official gene nomenclature where applicable, while indicating protein function. Commonly used synonyms are listed within an entry, but the recommended name is that which describes the protein to the fullest extent. The guidelines followed to generate the recommended protein name are available at https://ftp.uniprot.org/pub/databases/uniprot/current_release/knowledgebase/complete/docs/International_Protein_Nomenclature_Guidelines.pdf. Gene names are separately added by the appropriate genome nomenclature committees, but UniProt curators are in constant communication with these groups to keep naming consistent, where possible.

## Functional Annotation

MS proteomics experiments generate lists of peptides which are subsequently mapped to proteins, and scientists are generally looking to link sets of these proteins to a common function, or set of functionalities, in order to understand a particular biological or biomedical observation. UniProt curators describe the activities of a protein, synthesizing brief summary descriptions from the vast array of experimental data scattered across the biomedical literature. These descriptions are collated under domain-specific headings, such as catalytic activity, activity regulation, tissue specificity, and subcellular location, and were originally almost completely free-text, making the analysis of large protein datasets extremely difficult. Increasingly, in order to better serve user groups such as the proteomics community, these data are being converted to controlled syntax statements and/or mapped to controlled vocabularies and ontologies enabling computational searching, data merger, and data analysis. Structured fields which are already fully computationally accessible include UniProt keywords, a hierarchical controlled vocabulary which summarizes the content of a UniProtKB entry, describing biological process, cellular component, coding sequence diversity (*e.g.*, alternative initiation, alternative promoter usage), developmental stage, disease, domain, ligand, molecular function, PTM, and technical term (*e.g.*, direct protein sequencing). UniProtKB Subcellular location and Disease sections are also described using fully documented controlled vocabulary terms. Catalytic activities in UniProtKB are annotated using Rhea, a reference vocabulary of biochemical reactions (https://www.rhea-db.org) (23). Rhea uses the chemical ontology ChEBI (18) to describe reaction participants, their chemical structures and chemical transformations, and all molecules described in the Cofactor section are also mapped to the ChEBI ontology.

Functional annotations for specific isoforms and polyprotein cleavage products are captured when these differ from those of the canonical reference protein in an entry. These are stored separately in the record to enable computer accessibility, and the representation of these data has been enhanced on the UniProt website (Fig. 2).

For a fully computationally accessible description of the molecular function(s) of a protein, the process(es) in which it plays a role and the cellular component(s) it is located in, most researchers use the Gene Ontology (GO) (24), a machine-readable ontology of terms which can be mapped to proteins and enables the computational analysis of large-scale experiments in biomedical research. UniProtKB curators create a significant proportion of the manual GO annotations generated each year, in particular for human proteins, and other UniProt annotations (*e.g.*, subcellular locations, EC number assignments, keywords) are used for computational mappings of terms to less well-studied proteins. All annotations (currently for >160 million proteins) are linked to UniProtKB identifiers by the GO Annotation team and available in this form for download from the QuickGO database (www.ebi.ac.uk/QuickGO/) and are also visible in the relevant UniProtKB records.

## Protein Structures

UniProtKB provides residue-level mappings between entries from PDB and the corresponding sequences in UniProtKB, with a dedicated UniProt curator manually linking cases where the sequence has mutated beyond the point where a computational match can be made (25). This invaluable dataset has been recently supplemented by the AlphaFold structural predictions (26), initially covering the complete proteomes of 21 species, but extended to a large proportion of all catalogued proteins in the UniProt, UniRef90 clusters—clustered sets of sequences from UniProtKB (including isoforms) and selected UniParc records which represent complete coverage of the sequence space at 90% sequence identity. UniProt thus enables the proteomics scientist to link sequence, structure, and function for proteins of interest.

## Visualization of Sequence Features and Structural Annotations

The ability to visualize a number of different sequence features co-occurring on the same regions of a protein sequence can give the user important insight into the mechanism of action of a particular protein or an understanding of how a disease variant causes a change in the function of that protein. For example, a disease variant may occur in the active site of an enzyme, suggesting the change in amino acid may result in a change or loss of catalytic activity. An experimentally induced positional mutation may confirm the existence of a PTM, give an indication of protein function or cellular/organism phenotype observed as a result of that change. Mapping of that feature to a 3D structure, experimentally generated or predicted, may give additional information as to the effect of a change to the protein sequence or by a chemical modification caused by a PTM. The ProtVista viewer (27) has been created by the UniProt development team to enable the graphical representation of protein sequence features in UniProtKB and align these with large-scale public datasets generated by, for example, proteomics or variation sequencing experiments. These are all displayed as separate tracks, mapped to the full length of the protein sequence. Additional data, such as the predicted deleterious effect of an amino acid variant (as calculated using Sorting Intolerant From Tolerant or Polymorphism Phenotyping) can be added as a filter and/or used to color-code the output. Customizable tooltips can be used to provide additional information. The protein 3D structure, experimental or predicted, can be shown in the same view, and the position of selected features are highlighted on the structural representation. This will enable the significance of features observed in a proteomics experiment, such as potential PTMs, to be further understood in the context of protein tertiary structure and topology. ProtVista is built using the Nightingale library of re-usable web components which are available for download and local installation (https://github.com/ebi-webcomponents/protvista-uniprot). Many of these described features are now also directly embedded into the entry page.

## What does the MS Community Provide to UniProt?

In addition to supporting the MS community through annotation of protein sequence and function, UniProt additionally accesses data from public domain proteomics data repositories and integrates proteomics data through a series of peptide import pipelines, using this information to inform and enhance relevant entries.

### Integrating Proteomics Repository Data into UniProtKB

**Fig. 6:**
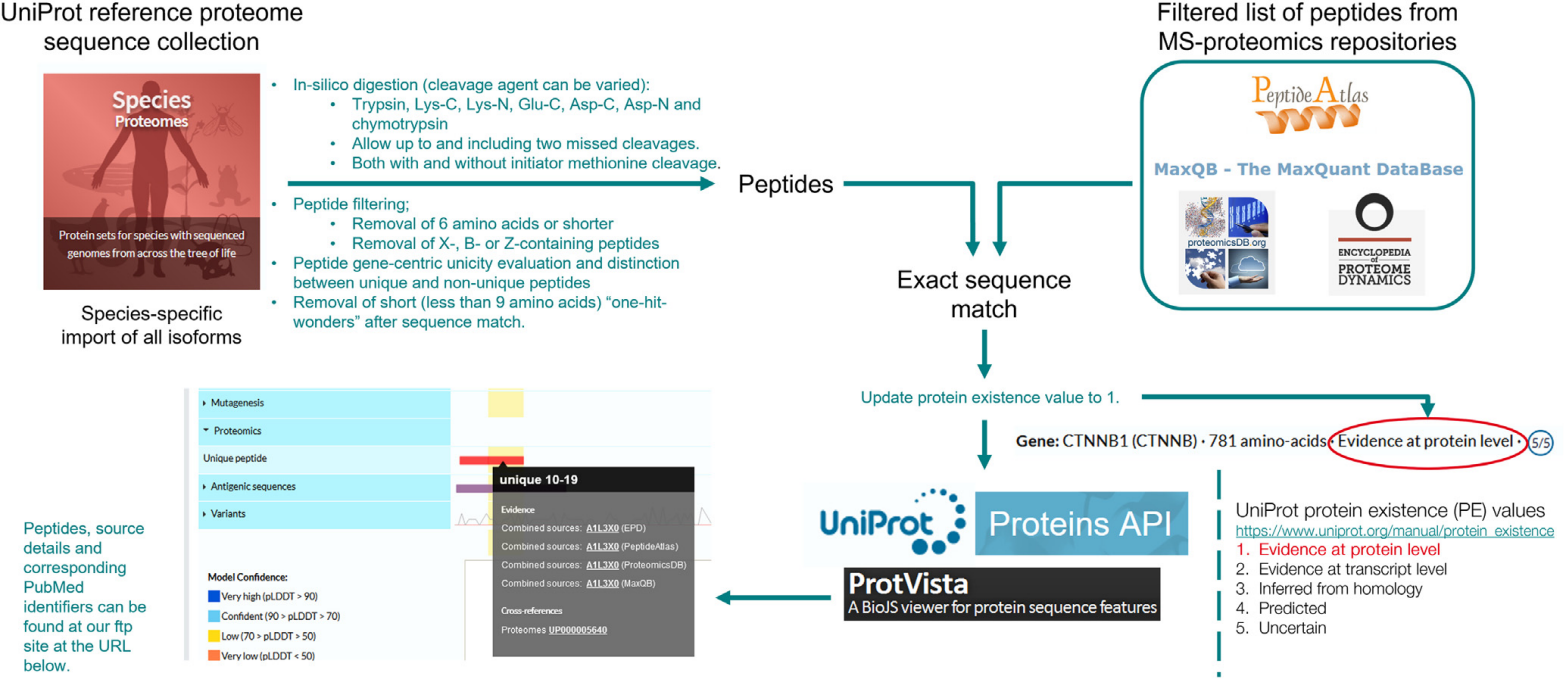
**Import pipeline of public proteomics data into UniProtKB.** Unreviewed UniProtKB/TrEMBL entries that are uniquely identified by a peptide have their Protein existence level set to 'Experimental evidence at protein level'—*red circle*.

**Fig. 7:**
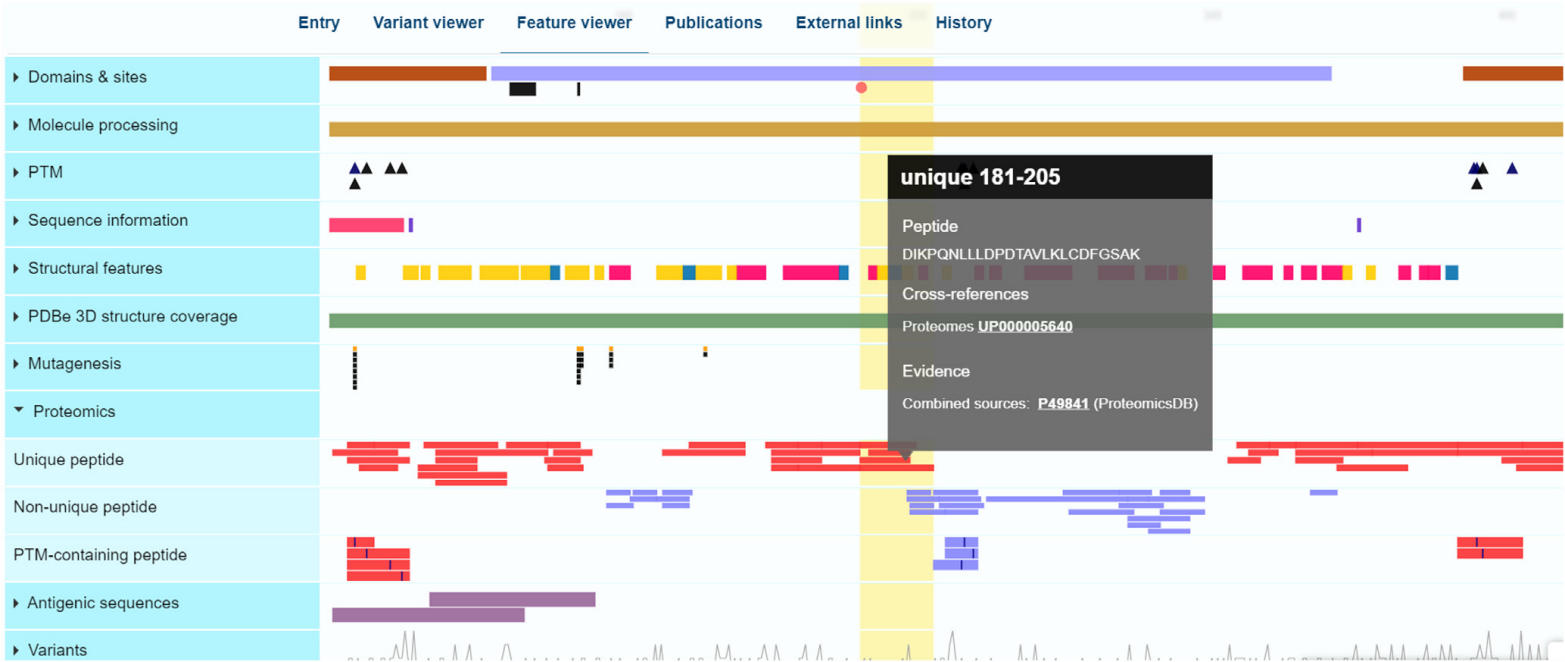
**Unique mapped peptide imported *via* the UniProt proteomics pipeline (****https://www.uniprot.org/uniprotkb/P49841****) shown in the feature viewer**. The Proteomics track is expanded, and unique peptide information details are further expanded.

UniProt cross-references to an increasing number of proteomics databases, including PRIDE (28), PeptideAtlas (29), CPTAC (30), jPost (31), MassIVE (32), MaxQB (33), PaxDB (34), and ProteomicsDB (35), many of which are members of the ProteomeXchange Consortium (32). These links enable users to access additional data on their protein of interest, for example to see in which tissues evidence of protein expression has been observed at the peptide level. Further to this, UniProt has developed a pipeline to analyze datasets from selected public MS-based proteomics resources. These resources provide tools for processing sequence and spectral data from publicly deposited proteomics experiments and UniProt bioinformaticians, with expertise in proteomics, work collaboratively with them to identify high-quality peptides that are then extracted and mapped to UniProtKB sequences (Fig. 6). Mapped peptides are taken as experimental evidence of the existence of the corresponding proteins. An unreviewed UniProtKB entry whose gene is uniquely identified by a peptide is annotated with the keyword ‘Proteomics identification’, and the Protein existence level is set to 'Experimental evidence at protein level'. These data are currently not used to annotate reviewed UniProtKB entries. Unique and nonunique peptides, mapped to the underlying protein sequence are visualized in the Proteomics track of the Feature viewer (Fig. 7) and can be accessed *via* the proteomics endpoint in the Proteins application programming interface (API). This pipeline is currently under review, with the intention of updating the quality metrics to bring these more in line with those published by the Human Proteome Project (36)

### PTM Data from Public Mass Spectrometry-Based Proteomics Resources

**Fig. 8:**
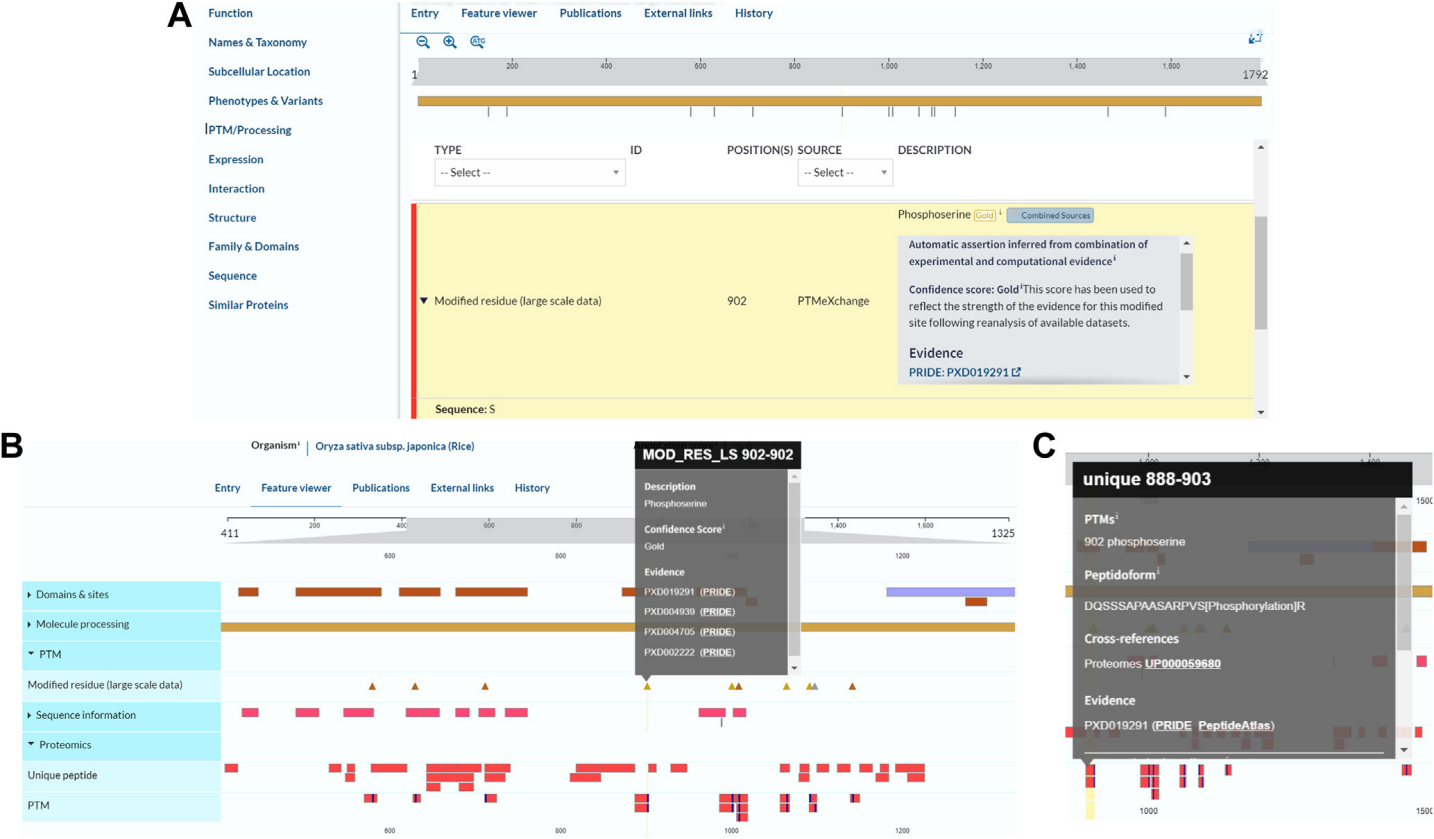
**PTM data imported *via* the PTMeXchange project in the protein entry page (****https://www.uniprot.org/uniprotkb/B9FXV5****) visualized by ProtVista**. *A*, Data can be filtered by type and/or source *B*, evidence tags expand to show confidence scores and *C*, links to associated datasets in PRIDE and PeptideAtlas. PTM, posttranslational modification.

The PTMeXchange project aims to establish a reproducible pipeline to import large-scale PTM-focused proteomics datasets. This collaborative project between UniProt, PRIDE, PeptideAtlas, and the University of Liverpool facilitates filtering, re-analysis, and site-based confidence scoring of PTM-specific public proteomics datasets from significant model organisms (37). Filtering and reanalysis ensure that only high-quality datasets are imported and reanalyzed, with these datasets stored in PRIDE attributed a unique ID (PXD), thereby facilitating user access, traceability, and reusability. Modified sites are assigned a confidence score based on their false localization rate across multiple datasets to reflect the strength of evidence available. Data are integrated into UniProtKB and visualized in ProtVista both in the PTM/Processing section of the protein entry page (Fig. 8) and in the feature viewer in site-centric format. Modified peptide data are visualized in the proteomics section of the feature viewer, and both site-centric and peptide-centric data are accessible *via* the Proteins API. Currently, rice (*Oryza sativa* subsp. japonica) proteomic PTM data are available with plans for *Plasmodium falciparum*, *Arabidopsis thaliana*, mouse (*Mus musculus*), and human (*H. sapiens*) to follow shortly.

### High-Quality Binary Protein Interaction Data from IMEx Interaction Databases

**Fig. 9:**
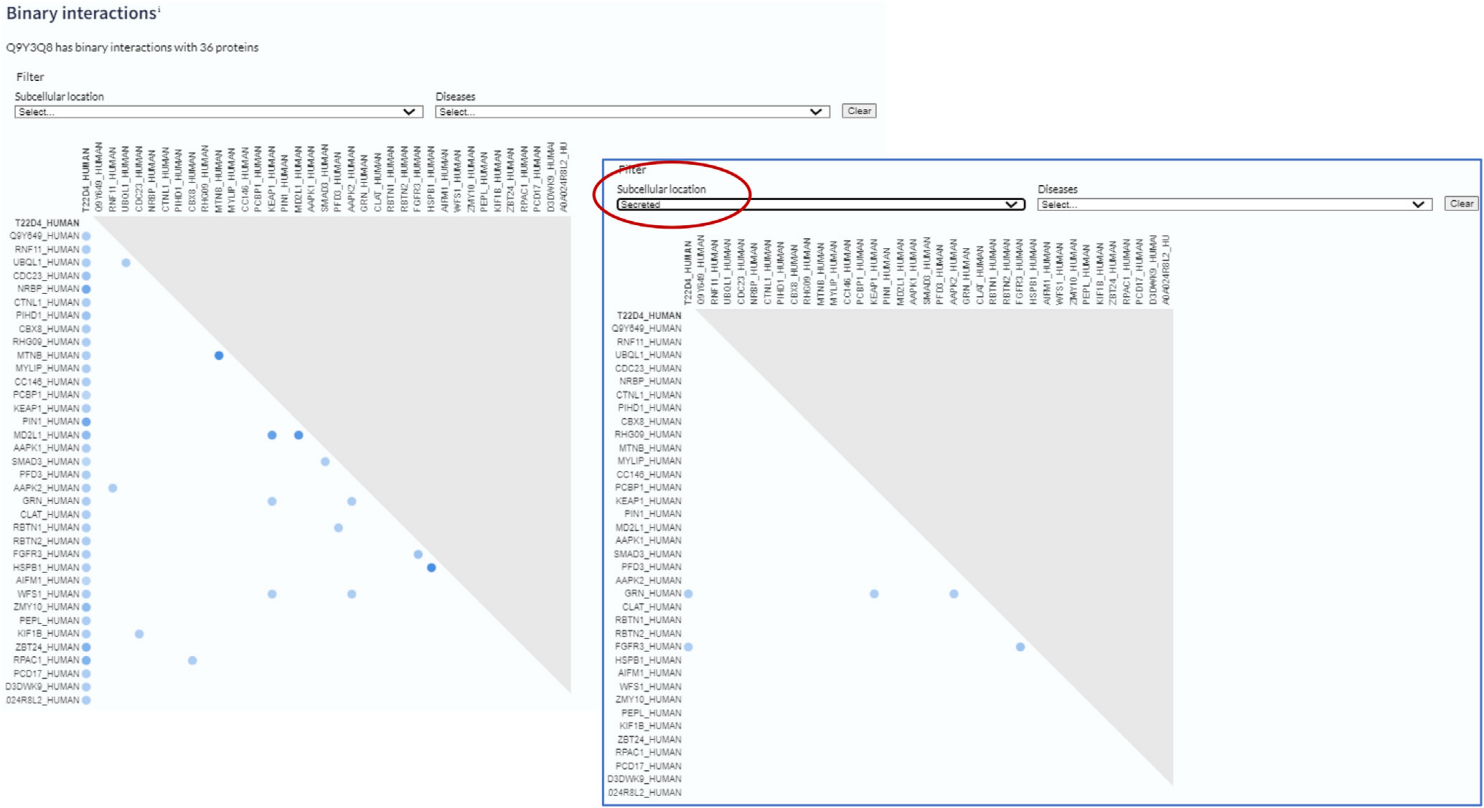
**Interaction subsection of a protein entry showing graphical representation of imported binary interactions of human SPACDR (****https://www.uniprot.org/uniprotkb/Q9Y3Q8****)**. Interactions can be filtered by subcellular location (shown in insert) and identification in disease.

Protein interaction data generated from affinity purified-MS is routinely captured by protein interaction databases. UniProt works with the IMEx Consortium (38), which combines this data with that generated by other methodologies (protein complementation assays, structural data, etc.) to generate concatenated binary interactions, which are scored, filtered, and a high-quality subset exported to UniProt for incorporation into the Interaction section of the relevant entries (Fig. 9). Users are able to visualize local networks of high-quality interactions using the Interaction adjacency viewer (https://github.com/ebi-uniprot/interaction-viewer) which is also available for local installation. Links to stable protein complexes curated by the Complex Portal (39) are already available in many entries, and additional information will be imported from this resource in 2023.

## Accessing Data

UniProtKB holds a wealth of data, and proteomics scientists need to be able to access this to select the subset, which is most appropriate to their needs, download in a format most correct for the use case (uploading into a search engine, local analysis etc), or to run specific, sophisticated queries *in situ*. In order to support this, UniProt has developed a number of access routes and supports multiple community-developed data formats.

### Data Formats

UniProtKB entries are available in various file formats including TSV, XML, RDF/XML, text, GFF, and JSON. UniProtKB entries download using these formats each contain only one protein sequence, the 'canonical' sequence. UniProtKB canonical sequences are also available in FASTA format, as are additional manually curated isoform sequences that are described in UniProtKB/Swiss-Prot. UniProt also provides the new format PEFF (PSI Extended FASTA Format), a unified format for protein and nucleotide sequence databases developed by the HUPO-PSI (Human Proteome Organization-Proteomics Standard Initiative) to be used by sequence search engines and other associated tools (*e.g.*, spectral library search tools). PEFF includes support for encoding known sequence variants, PTMs, and proteoforms and enables search engines implementing this format to query the exact positions of known PTMs and variants. The UniProt PEFF format currently describes variation and sequence data and is available through the Proteins API service (https://www.ebi.ac.uk/proteins/api/doc/) for 31 species. An extension to include PTM data is planned. UniProt personnel are also actively collaborating with the HUPO-PSI and the Consortium for Top Down Proteomics who are developing a notation format called ‘ProForma’ (40) aiming to represent proteoforms, *i.e.*, all possible variations of a protein/peptide sequence, including protein modifications and will make data available in this format when agreed upon.

### Accessing Data *via* the UniProt Website

To search, select, and download a set of protein entries appropriate to the experiment that the researcher is planning, it may be the simplest option to run queries *via* the website interface. The researcher will certainly wish to be familiar with this for subsequent searches on individual proteins or lists of proteins. The UniProt website (www.uniprot.org) enables access for approximately 800,000 users per month totaling nearly 9.5 million page views (November 2022), so needs to be reliable, responsive, and usable on all platforms. With this in mind, we regularly assess the existing website in terms of performance and have undertaken a full release of a new version in July 2022. This has been iteratively designed and created through a user-centered design process and now separates the front-end (Web user interface) from the back-end (API). This improves responsiveness while remaining scalable and eases maintenance. The new design includes improved search and navigation using a caching logic to support both simple and complex popular queries. Once a search has been completed, search results can be downloaded in predefined FASTA, XML, RDF/XML, and text formats and also a customizable tab-separated or Excel format (Fig. 1).

**Fig. 10:**
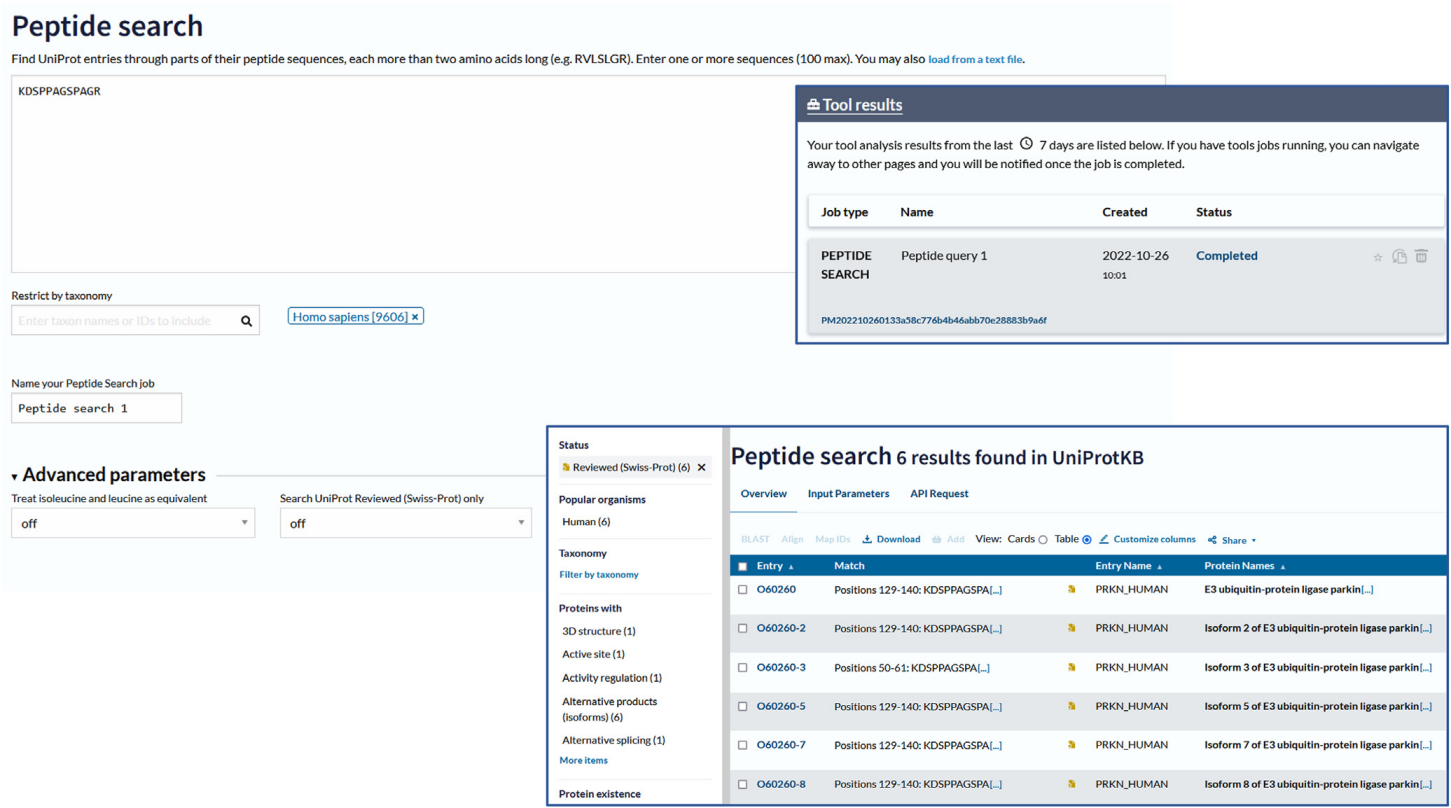
**The****UniProt.org****peptide search tool (****https://www.uniprot.org/peptide-search****) can be used to search for proteins that map to query peptides, search is restricted to Human (TaxID; 9606).** Peptide search jobs are run and stored in the tools dashboard, and results show mapping of the query peptide to human Parkin isoform entries restricted to Reviewed (UniProtKB/SwissProt) protein entries.

Resources accessible through the website specifically designed to assist MS–based research include the previously described Proteomes collections, where a dedicated facility enables the search and download of proteomes. Additionally, users of the Peptide search can enter a peptide sequence (for example from a proteomics experiment) into the search field and the tool quickly finds all UniProtKB sequences that match 100% with the query sequence (Fig. 10). Searches can be restricted to a taxonomic subset of UniProtKB to further decrease the search time. Future plans for this resource include the extended display of the matched region in context with adjacent sequences, links to peptides at PRIDE (28), to identify datasets the peptide has been identified in, and Immune Epitope Database (41) and also implementation of a batch search option. We will add a new option to allow some ambiguity in a peptide to match similar but not identical peptides. This option will be useful in searching for variation related to immune epitopes, neoantigens, and functional domains.

### Accessing UniProtKB Data Programmatically

**Fig. 11:**
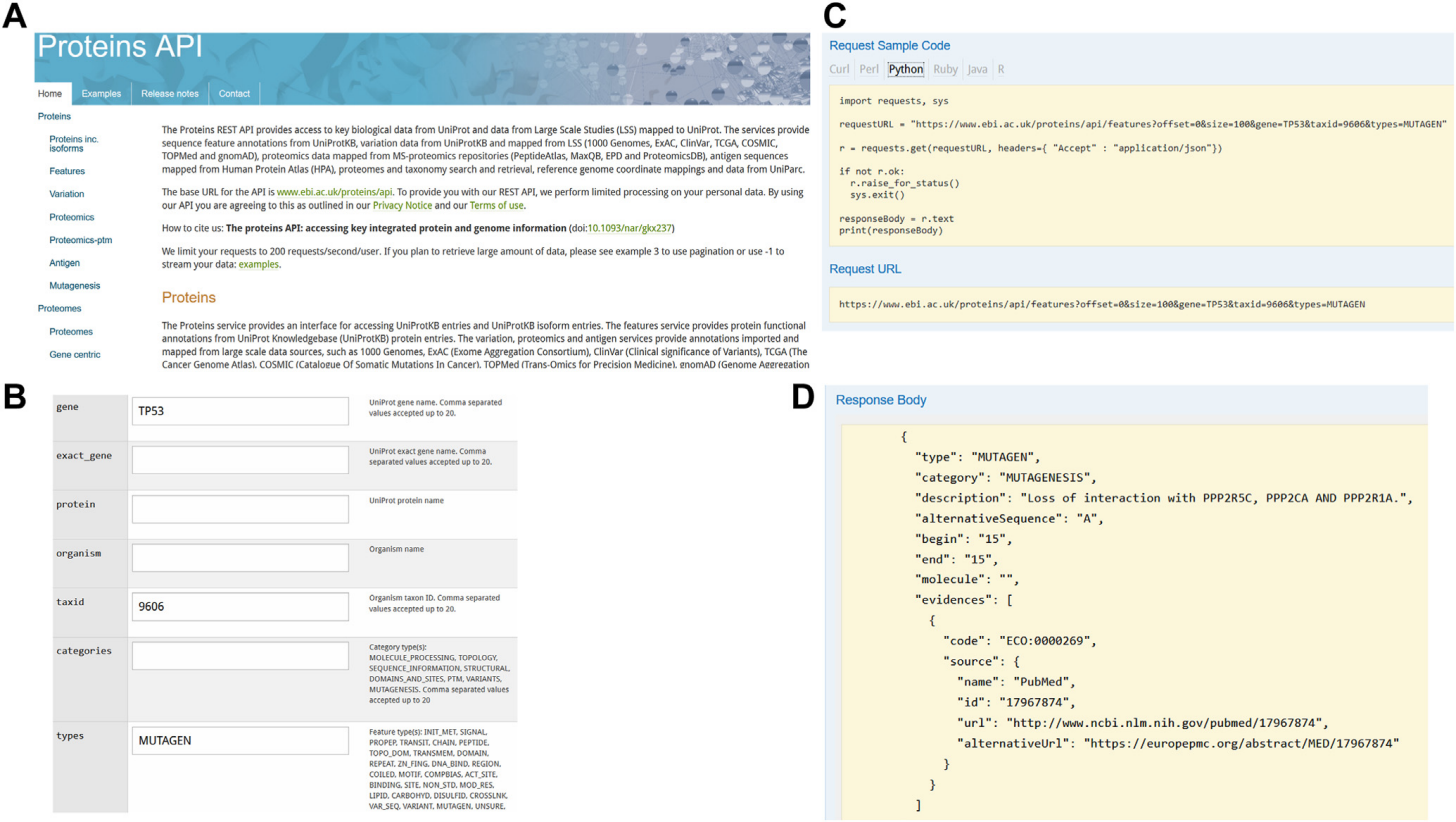
**UniProt Proteins API homepage provides access to a range of database accessibility options.***A*, a toolbar on the right-hand side of the page list these options. *B*, each subsection of the API has a multiquery form to allow building of complex filtered queries, in this case in the features subsection a search for ‘mutagens’ in the gene ‘TP53’ restricted to Human ‘9606’. *C*, each query provides example code in a range of languages and a URL to allow access to the results in a webpage. *D*, the ‘Response Body’ provides the results of the query, ordered by amino acid residue number. API, application programming interface.

The UniProtKB Proteins API (42) provides an intuitive, fast, easily accessible interface, so wet lab and computational scientists alike can gain access to protein sequence positional annotations and imported genomics and proteomics data, such as amino acid variations or peptide sequences. Additional data sources include antigen sequences mapped from Human Protein Atlas, proteomes and taxonomy search and retrieval, and reference genome coordinate mappings. The Representational state transfer (REST) API (https://www.ebi.ac.uk/proteins/api/doc/) (Fig. 11*A*) does not require users to be fluent in a programming language and instead provides a multiquery search format (Fig. 11*B*) that allows users to intuitively query the database and return a readable and detailed response to queries. Users can tailor their search queries within the multiquery search form sections to restrict their search criteria by categories such as species, genomic location, peptide uniqueness, or even by source database. Following each query, a sample request code in multiple programming languages, such as Java, R, and Python, is provided that gives the user a programmatic query with which to recreate their search *via* their own desktop terminal (Fig. 11*C*). Additionally, for those not looking to recall their search results programmatically, a URL is provided that directs them to their results on a webpage. All data provided by the Proteins API are freely downloadable in a range of formats (Fig. 11*D*). Data derived from sources external to the UniProtKB are currently only available programmatically through the service.

UniProt website RESTful URLs can be bookmarked, linked, and used in programs for all entries, queries and tools available through the website and a Java library is also provided that enables a stable remote API for programmatically accessing UniProt data.

### Accessing Data *via* SPARQL

All UniProt data are available in RDF (Resource Description Framework), a core semantic web technology which enables researchers to perform analytic query operations without knowing the underlying database structure. The UniProt SPARQL service (https://sparql.uniprot.org) allows users to perform complex federated queries that combine RDF data from UniProt with that from other SPARQL endpoints. For example, it is possible to query for ligand-binding site annotations in UniProtKB using identifiers, names, synonyms, and chemical structure data from ChEBI.

### Accessing Data *via* FTP

The UniProt FTP site (accessible *via* the ‘Download latest release’ link located on the UniProt.org homepage) provides the most frequently requested datasets in a range of file formats (Flat Text, XML, RDF/XML, and FASTA). The additional manually curated isoform sequences that are described in the UniProtKB/Swiss-Prot section are available in a separate FASTA file (uniprot_sprot_varsplic.fasta.gz). Our FTP directory also includes expanded FASTA sets, containing both the canonical and manually reviewed isoform sequences, for all reference proteomes.

## Conclusions

UniProt plays a pivotal role in support of MS proteomics science, collecting, standardizing, and organizing protein sequence data and creating a reference framework for multiscale data integration and analysis. From the provision of FASTA and PEFF files at the whole proteome level which enables search engines to operate to enabling the annotation of proteins with biological function, UniProtKB is a crucial data management tool, working with the community to ensure the best possible service is supplied. Members of the UniProt team actively collaborate with proteomics standards bodies, including HUPO and the HUPO-PSI, to both input into the development of standards, formats, and controlled vocabularies and to implement these in UniProt whenever appropriate. Additionally, UniProtKB is working with data resources such as the members of the ProteomeXchange, PTMeXchange, and the IMEx Consortium to develop data import pipelines, enabling the output of the MS proteomics community to inform and enhance protein sequence and function data.

Large-scale data are an asset when converted to knowledge (43) and the work of the UniProt database empowers proteomics scientists to achieve this, both as a sequence reference resource and as a knowledgebase of protein function. Researchers need to spend only a small fraction of the time otherwise required to read and evaluate literature data, because of the work of the UniProt expert curators. By integrating proteomics data back into the UniProt database, UniProt helps to facilitate data dissemination, while ensuring links to the original datasets and repositories are maintained. Researchers are encouraged to make full use of the richness of UniProt data to help support proteomics data standards and deposit data into the public domain and to work with us to enhance the invaluable resource that is UniProt.

## Conflict of interest

The authors declare no competing interests.
